# Avoiding Diagnostic Delays in Neonatal Gastric Volvulus: A Rare Case With a Favorable Outcome

**DOI:** 10.7759/cureus.102677

**Published:** 2026-01-30

**Authors:** Konstantine Kvaratskhelia, Tamar Gvalia, David Gagua, Tinatin Gagua, Tamar Khutchua

**Affiliations:** 1 Neonatology, Gagua Clinic, Tbilisi, GEO; 2 Obstetrics and Gynecology, David Tvildiani Medical University, Gagua Clinic, Tbilisi, GEO

**Keywords:** abdominal distension, contrast imaging, feeding intolerance, gastric volvulus, gastrointestinal obstruction, neonate, pediatric surgery, radiographic diagnosis

## Abstract

Neonatal gastric volvulus (GV) is a rare but potentially life-threatening condition caused by abnormal rotation of the stomach, leading to obstruction, ischemia, or perforation. Early diagnosis is challenging due to nonspecific initial symptoms and the limited diagnostic value of plain radiographs.

We present a case of late preterm GV in which the diagnosis was delayed until repeat contrast imaging was performed. Timely surgical referral following this led to a favorable outcome. A male infant was delivered vaginally at 36 + 3 weeks’ gestation, weighing 2200 g and measuring 44 cm, with Apgar scores of 7 and 8 at one and five minutes, respectively. Shortly after birth, he developed respiratory distress requiring mechanical ventilation in the NICU. Nasogastric tube placement revealed active gastric bleeding, prompting cryoplasma transfusion and initiation of parenteral nutrition. Reverse peristalsis, undigested gastric contents, and progressive abdominal distension followed attempts at minimal enteral feeding. Initial supine abdominal radiographs showed nonspecific gas distribution. On day 6, an upper gastrointestinal (GI) contrast study excluded intestinal obstruction but demonstrated delayed gastric emptying. Persistent symptoms led to a repeat contrast study, which revealed a spherical stomach with an air-fluid level, findings consistent with GV, prompting urgent surgical referral.

This case underscores the diagnostic limitations of plain radiography in neonatal GV and the risk of delayed recognition when contrast imaging is postponed. Persistent GI symptoms, particularly feeding intolerance and unexplained distension, should prompt early escalation to targeted imaging modalities. In this case, initial radiographs failed to identify GV, delaying diagnosis. The abnormal rotation and positioning of the stomach became evident only on a later upper GI contrast study, allowing for timely diagnosis and surgical intervention. This case highlights the importance of early contrast imaging in neonates with persistent, unexplained GI symptoms to prevent avoidable delays in care.

## Introduction

Neonatal gastric volvulus (GV) is a rare but potentially life-threatening surgical emergency involving abnormal rotation of the stomach along its long (organoaxial) or short (mesenteroaxial) axis. First described by Berti in 1866 and later defined by Borchardt in 1904, GV remains difficult to diagnose in neonates due to nonspecific clinical signs and the limited diagnostic value of plain abdominal radiographs [1].

Symptoms such as feeding intolerance, non-bilious vomiting, difficulty passing a feeding tube to the stomach, and abdominal distension can mimic more common neonatal gastrointestinal (GI) conditions, contributing to delays in recognition. Although plain radiographs are often the initial diagnostic tool, they rarely provide definitive evidence of GV. A contrast-enhanced upper GI series is considered the gold standard, as it can identify abnormal gastric rotation and positioning [2,3].

Timely diagnosis is critical to prevent ischemic complications or perforation. While chronic or intermittent GV may sometimes be managed conservatively, acute cases typically require prompt surgical intervention [4]. Given its rarity and variability of presentation, continued reporting of neonatal GV cases is essential to raise clinical awareness and promote early recognition.

## Case presentation

A male neonate was delivered vaginally at 36 weeks and 3 days of gestation after an uncomplicated pregnancy. He weighed 2,200 g, measured 44 cm, and had Apgar scores of 7 and 8 at one and five minutes, respectively. No perinatal complications were reported, and prenatal ultrasounds were unremarkable.

**Figure 1:**
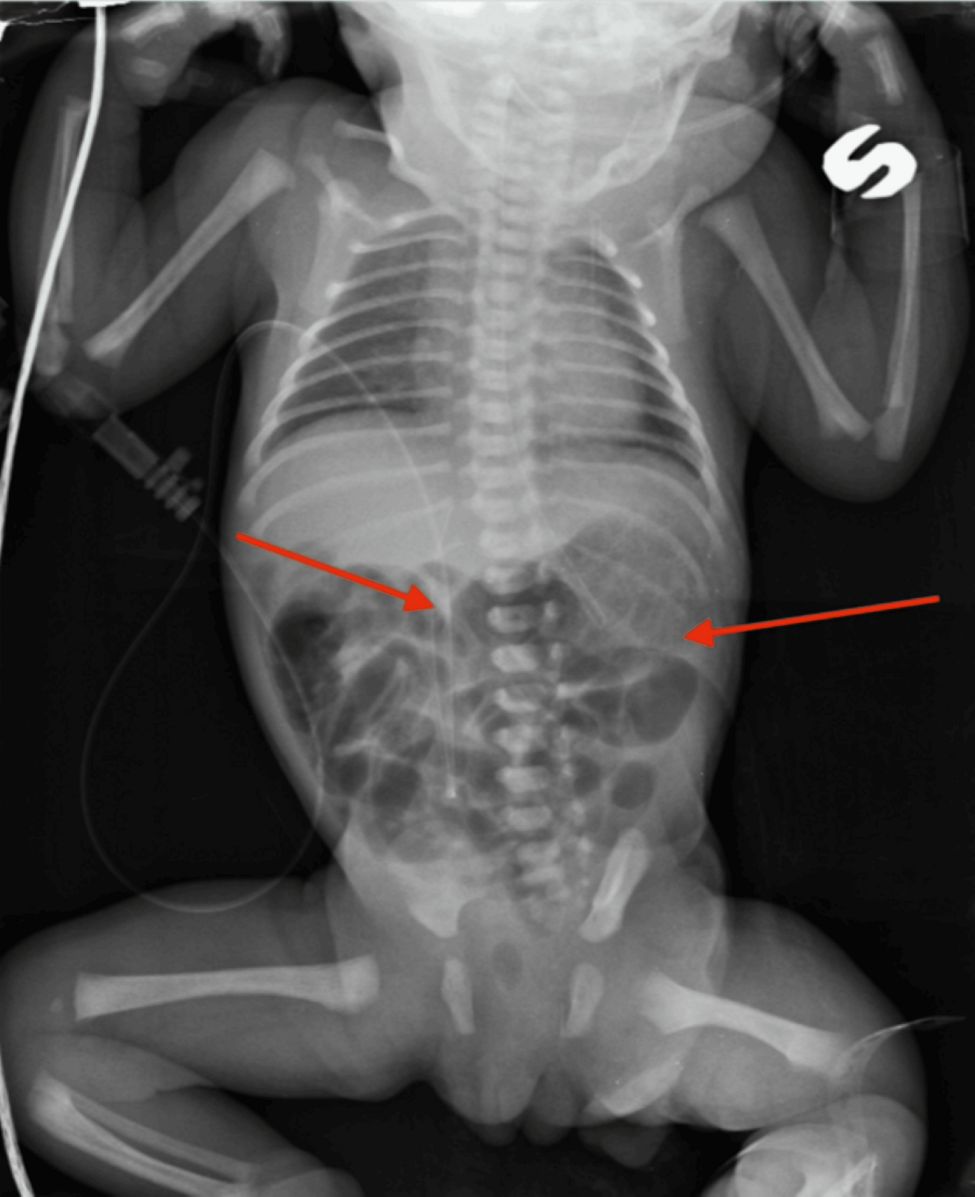
Initial supine chest-abdominal radiograph demonstrating mild gastric gaseous distension and scattered bowel gas (arrows), without clear radiographic evidence of obstruction.

Approximately three hours after birth, the infant developed progressive respiratory distress, manifested by tachypnea, subcostal retractions, and nasal flaring. Oxygen saturation dropped despite supplemental oxygen. Copious upper airway secretions further impaired ventilation, necessitating intubation and placement on synchronized intermittent mandatory ventilation (SIMV) (Figure 1).

Shortly thereafter, gastric bleeding was noted via nasogastric tube, with aspirates appearing bloody, prompting fresh-frozen plasma transfusion and initiation of total parenteral nutrition for stabilization. After stabilization, minimal enteral feeding was attempted. The infant exhibited feeding intolerance, with repeated reverse peristalsis, undigested gastric aspirates, and progressive abdominal distension, especially in the epigastric region. Neurologically, he remained lethargic. Serial infection workups, including blood cultures and inflammatory markers, were negative. Given the presence of feeding intolerance, early respiratory distress, and an abnormal GI gas pattern on initial radiography, tracheoesophageal fistula was included in the initial differential diagnosis and subsequently excluded by contrast imaging.

**Figure 2:**
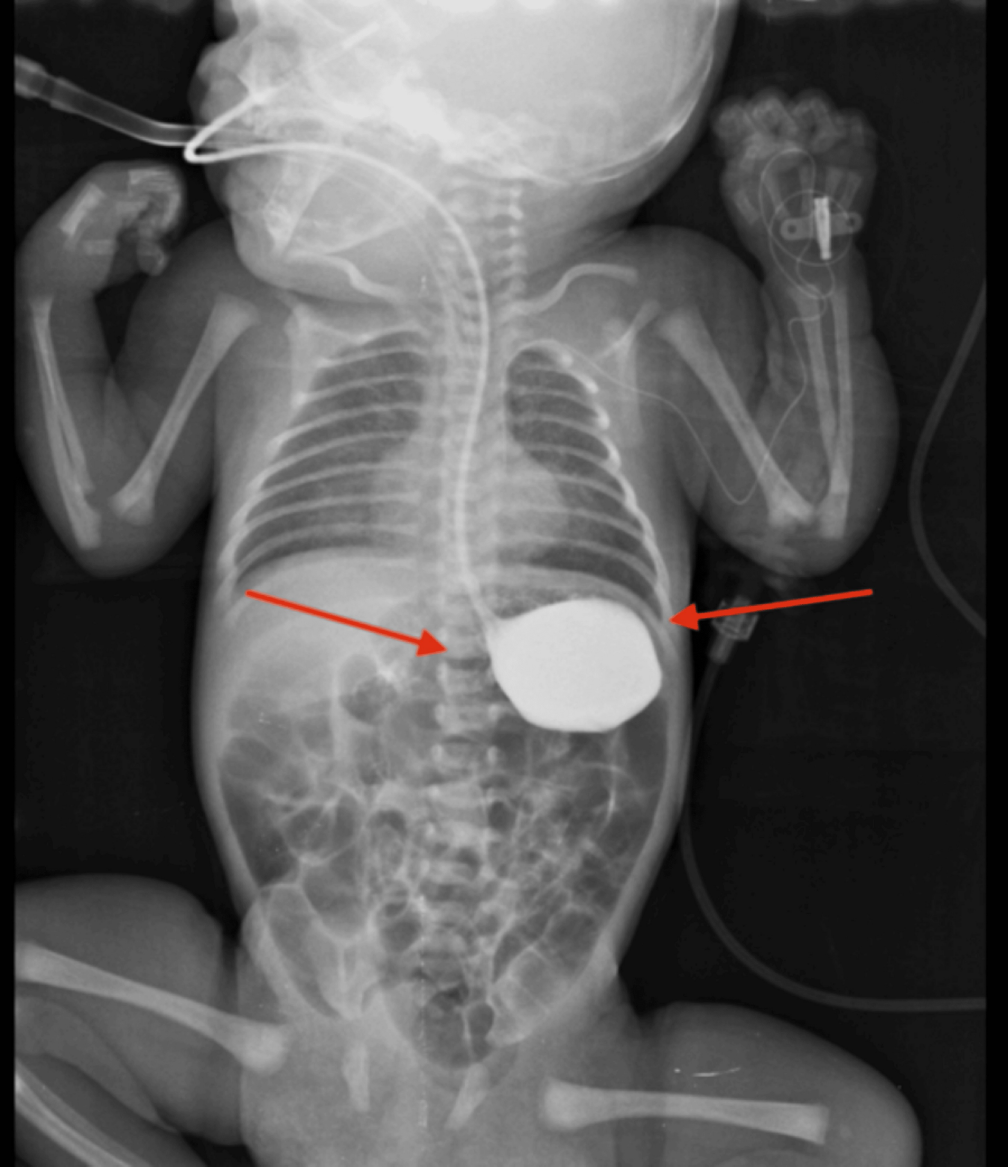
Day 6 contrast gastrointestinal study demonstrating delayed gastric emptying, with contrast retained in the proximal stomach but no evidence of volvulus (arrows).

On day 6, an upper GI contrast study was performed to evaluate ongoing feeding intolerance and abdominal distension. The study excluded intestinal obstruction, but demonstrated delayed gastric emptying with persistent contrast retention (Figure 2).

**Figure 3:**
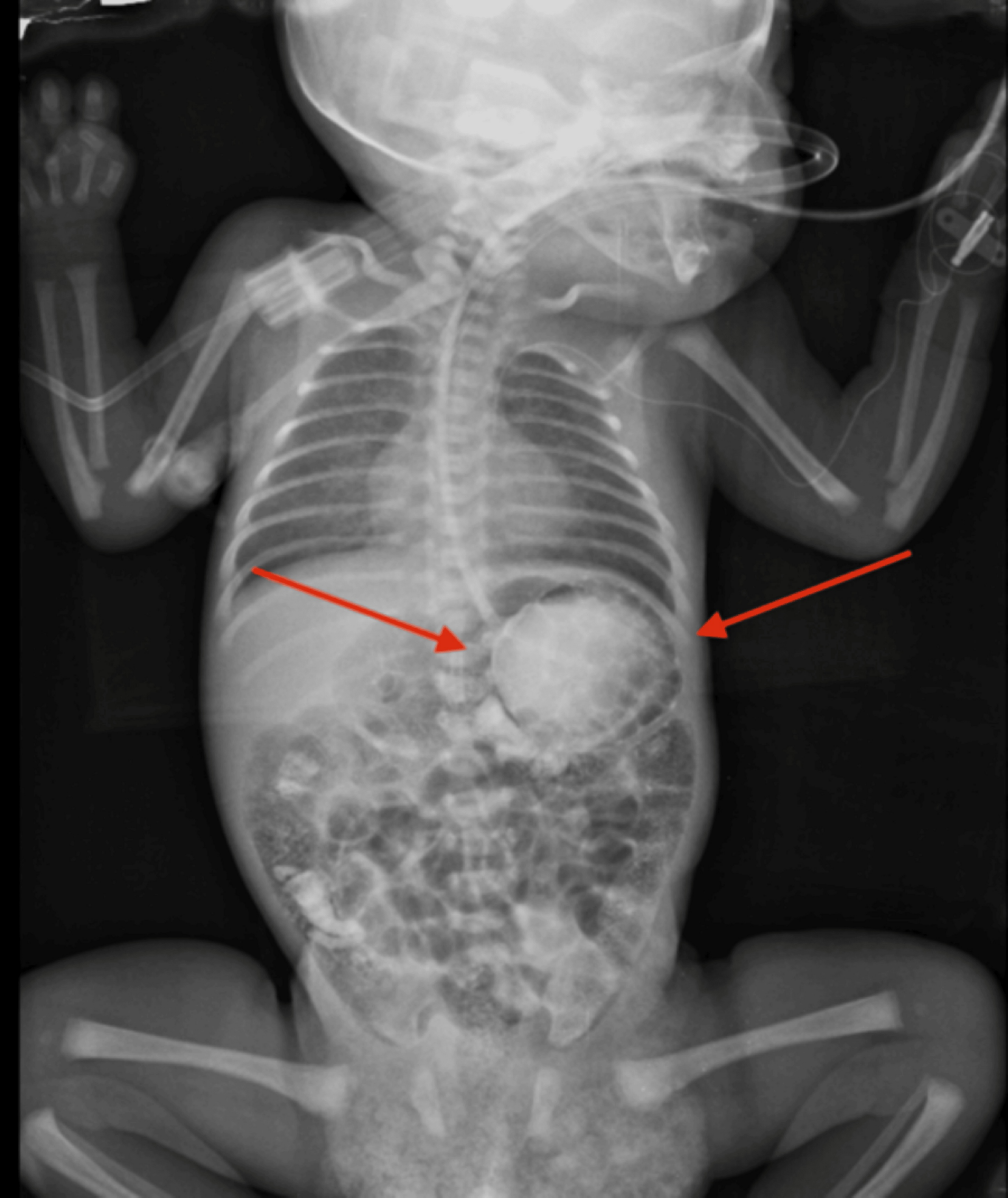
Pre-operative supine chest/abdominal radiograph follow-up imaging, showing persistent gastric distension despite decompression (arrows), correlating with ongoing feeding intolerance.

**Figure 4:**
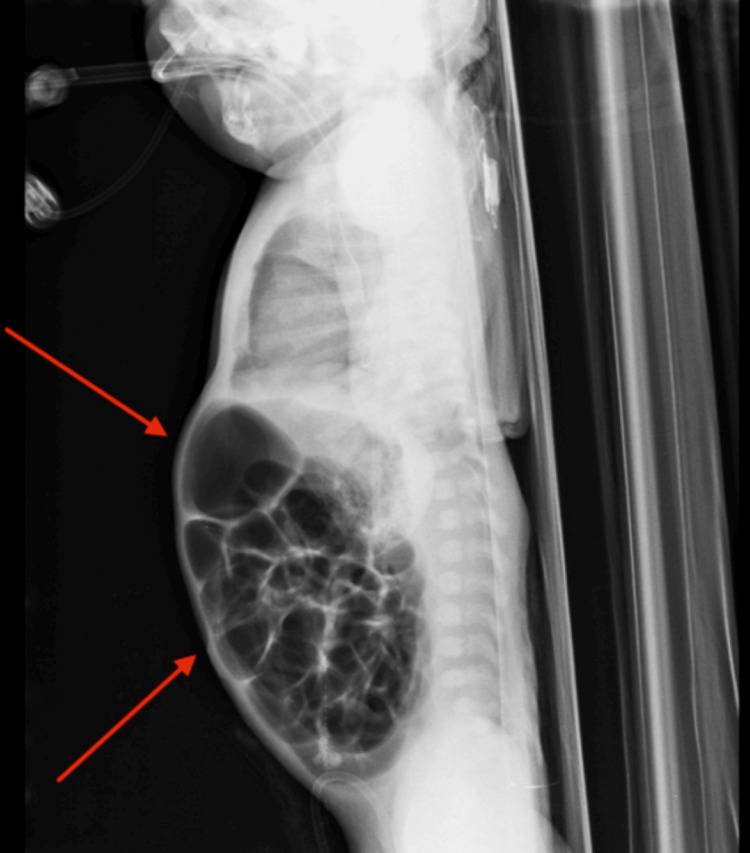
Pre-operative lateral chest/abdominal radiograph follow-up imaging, showing persistent gastric distension despite decompression (arrows), correlating with ongoing feeding intolerance.

Despite gastric decompression via nasogastric tube, the infant’s abdominal distension persisted, with visible epigastric fullness and intermittent episodes of vomiting (Figures 3-4).

**Figure 5:**
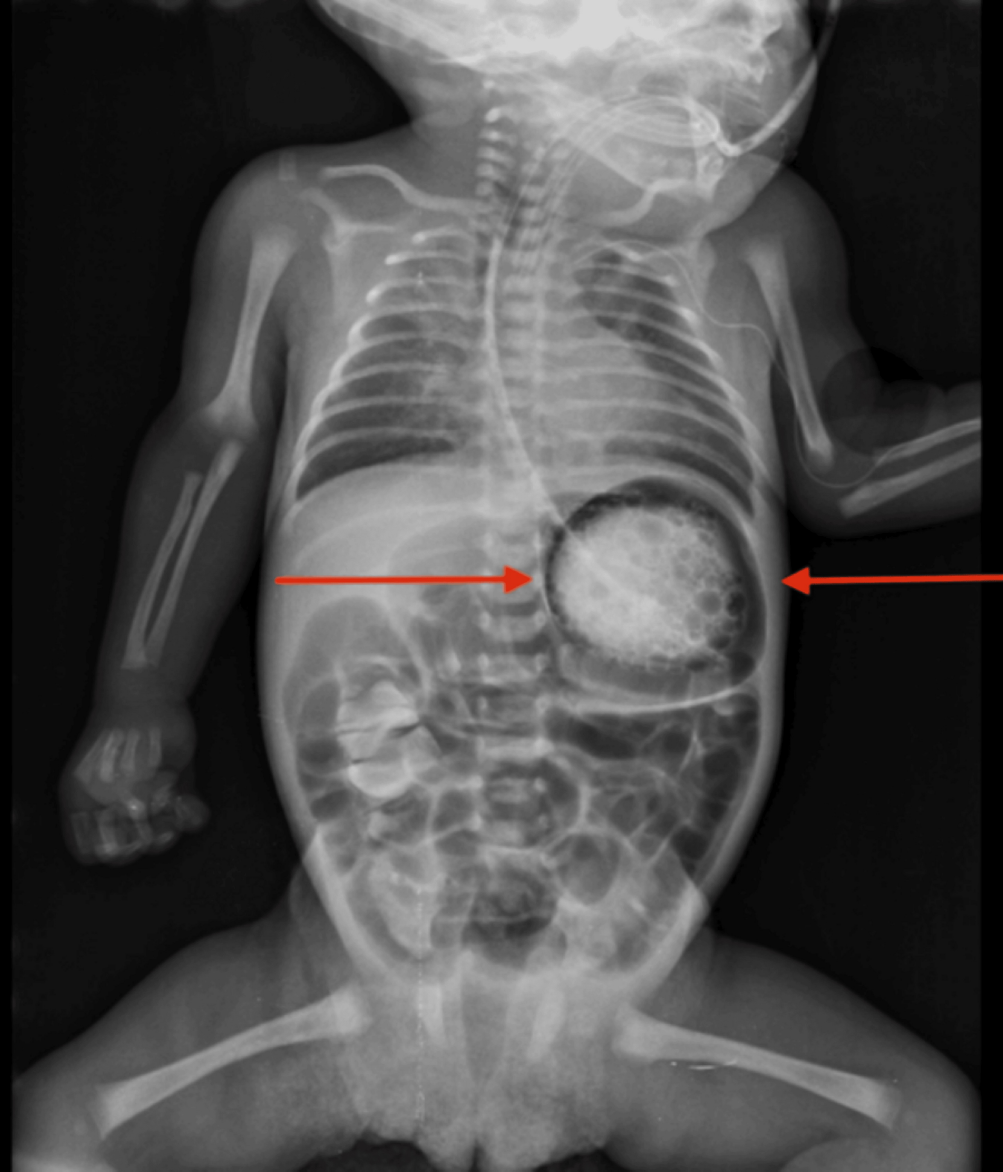
Repeat upper gastrointestinal contrast study demonstrating a spherical stomach with an air-fluid level and organoaxial rotation (arrows), consistent with gastric volvulus.

Given persistent symptoms, a repeat contrast study was obtained. This imaging revealed a spherical, abnormally positioned stomach with a prominent air-fluid level, findings consistent with GV (Figure 5).

The stomach appeared rotated along its long axis, with delayed passage of contrast into the duodenum. These findings prompted urgent surgical consultation and transfer for operative management.

The patient underwent an upper transverse laparotomy. Intraoperatively, the stomach was confirmed to be organo-axially rotated, but showed no evidence of ischemia or necrosis. The volvulus was corrected, and the pyloro-antral region was anchored to the anterior abdominal wall via anterior gastropexy, using a conventional open surgical approach to prevent recurrence. Postoperatively, the infant demonstrated rapid clinical improvement: feeding tolerance normalized, abdominal distension resolved, and he gradually transitioned to full enteral feeds. He was discharged home in stable condition, without complications. Follow-up at three months showed no evidence of recurrence.

## Discussion

Neonatal GV is a rare but serious condition that requires a high index of suspicion for timely diagnosis and intervention. Due to overlapping features with more common neonatal conditions - such as tracheoesophageal fistula or bowel atresia - GV often goes unrecognized until symptoms persist or worsen [1].

Plain abdominal radiographs are frequently inconclusive, as was the case in our patient. Contrast-enhanced upper GI studies remain the most reliable diagnostic tool for GV, particularly in detecting abnormal gastric rotation or delayed emptying [2,3].

Several case reports highlight the importance of repeat or early contrast imaging in patients with persistent GI symptoms. Kim et al. [3] described two neonates diagnosed with GV only after repeat imaging revealed organoaxial volvulus, despite earlier inconclusive studies. Similarly, Puspitasari and Setiawan reported a case of GV in a neonate with omphalocele, in which adhesions were found during surgery, after imaging failed to clarify the diagnosis [5]. El Azzouzi also described successful early diagnosis and surgical correction of intrathoracic GV related to congenital diaphragmatic hernia [6].

Additional evidence comes from a case reported by Long et al., in which a neonate with vomiting and poor weight gain underwent an early contrast study, revealing mesenteroaxial GV. Early surgery resulted in a favorable outcome without complications [7]. Furthermore, Inanc et al. reported a retrospective series supporting conservative management in chronic or intermittent GV, provided that the diagnosis is made promptly and symptoms remain stable [4].

Imaging findings in our case were consistent with previously reported features of neonatal GV, including a spherical, abnormally positioned stomach with an air-fluid level [8]. Other potential diagnoses were excluded based on clinical evaluation and investigations. The patient’s acute-onset, non-bilious vomiting, abdominal distension, and feeding intolerance, together with normal laboratory results, made infectious or metabolic causes unlikely. Abdominal X-ray and contrast study confirmed organo-axial GV and ruled out malrotation, pyloric stenosis, and intestinal atresia, supporting the final diagnosis [8].

This case highlights the importance of clinical persistence when symptoms persist despite standard care. Early use of targeted contrast imaging - and its repetition when needed - can prevent serious complications. Continued reporting of such rare presentations can help improve recognition, refine diagnostic algorithms, and reduce preventable delays.

## Conclusions

In this case, initial supine abdominal radiographs demonstrated only nonspecific gas patterns and failed to reveal GV, resulting in a delay in diagnosis. Definitive findings were obtained only after a repeat upper GI contrast study, which identified abnormal rotation and positioning of the stomach. This enabled timely surgical referral and intervention before the development of ischemic complications.

This case underscores the importance of early, and when necessary, repeated contrast imaging in neonates with persistent, unexplained GI symptoms. Prompt recognition of GV is essential to avoid preventable delays in care and to improve clinical outcomes in this rare but potentially life-threatening condition.
